# Prediction of optimal contrast times post-imaging agent administration to inform personalized fluorescence-guided surgery

**DOI:** 10.1117/1.JBO.25.11.116005

**Published:** 2020-11-16

**Authors:** Negar Sadeghipour, Aakanksha Rangnekar, Margaret R. Folaron, Rendall R. Strawbridge, Kimberley S. Samkoe, Scott C. Davis, Kenneth M. Tichauer

**Affiliations:** a Illinois Institute of Technology, Department of Biomedical Engineering, Chicago, Illinois, United States; b Stanford University School of Medicine, Molecular Imaging Program at Stanford, Palo Alto, California, United States; c Stanford University School of Medicine, Canary Center at Stanford for Cancer Early Detection, Palo Alto, California, United States; d Dartmouth College, Thayer School for Engineering, Hanover, New Hampshire, United States

**Keywords:** fluorescence-guided surgery, intraoperative visualization, optimal time of surgery, paired-agent imaging, kinetic modeling

## Abstract

**Significance:** Fluorescence guidance in cancer surgery (FGS) using molecular-targeted contrast agents is accelerating, yet the influence of individual patients’ physiology on the optimal time to perform surgery post-agent-injection is not fully understood.

**Aim:** Develop a mathematical framework and analytical expressions to estimate patient-specific time-to-maximum contrast after imaging agent administration for single- and paired-agent (coadministration of targeted and control agents) protocols.

**Approach:** The framework was validated in mouse subcutaneous xenograft studies for three classes of imaging agents: peptide, antibody mimetic, and antibody. Analytical expressions estimating time-to-maximum-tumor-discrimination potential were evaluated over a range of parameters using the validated framework for human cancer parameters.

**Results:** Correlations were observed between simulations and matched experiments and metrics of tumor discrimination potential (p<0.05). Based on human cancer physiology, times-to-maximum contrast for peptide and antibody mimetic agents were <200  min, >15  h for antibodies, on average. The analytical estimates of time-to-maximum tumor discrimination performance exhibited errors of <10% on average, whereas patient-to-patient variance is expected to be greater than 100%.

**Conclusion:** We demonstrated that analytical estimates of time-to-maximum contrast in FGS carried out patient-to-patient can outperform the population average time-to-maximum contrast used currently in clinical trials. Such estimates can be made with preoperative DCE-MRI (or similar) and knowledge of the targeted agent’s binding affinity.

## 1 Introduction

The extent or completeness of tumor tissue resection is correlated with median time to tumor progression and median survival for patients in a number of cancer types.^1^^–^^3^ The success of fluorescence-guided cancer surgery (FGS) relies on achieving a sufficient level of contrast between tumor and normal tissue such that a surgeon is able to better identify and remove more diseased tissue, perform the removal quicker, and minimize damage to healthy structures. For exogenously administered fluorescent agent protocols, the temporal progression of tumor-to-normal contrast depends on several characteristics of the targeted imaging agent and the tumor and healthy tissue physiology. Current approaches that select generalized dosing and imaging protocols for exogenous FGS based on experimental optimization have led to promising early clinical studies.^4^^,^^5^ However, physiological variability among and within patient cancers^6^^,^^7^—which encompasses parameters such as blood flow, vascular permeability, extent of vascularization, efficacy of lymphatic drainage, and cellular internalization and metabolism of the imaging agent—suggests that no single dosing and imaging protocol may be optimal for all patients. Furthermore, experimental (trial and error) optimization can be time consuming and costly, especially considering the abundant array of novel fluorescent imaging agents that are poised for clinical testing.^8^^–^^11^ Both of these limitations can be ameliorated by the development of accurate mathematical models capable of incorporating imaging agent characteristics and tissue physiological parameters to predict tumor-to-normal tissue contrast.

**Fig. 1:**
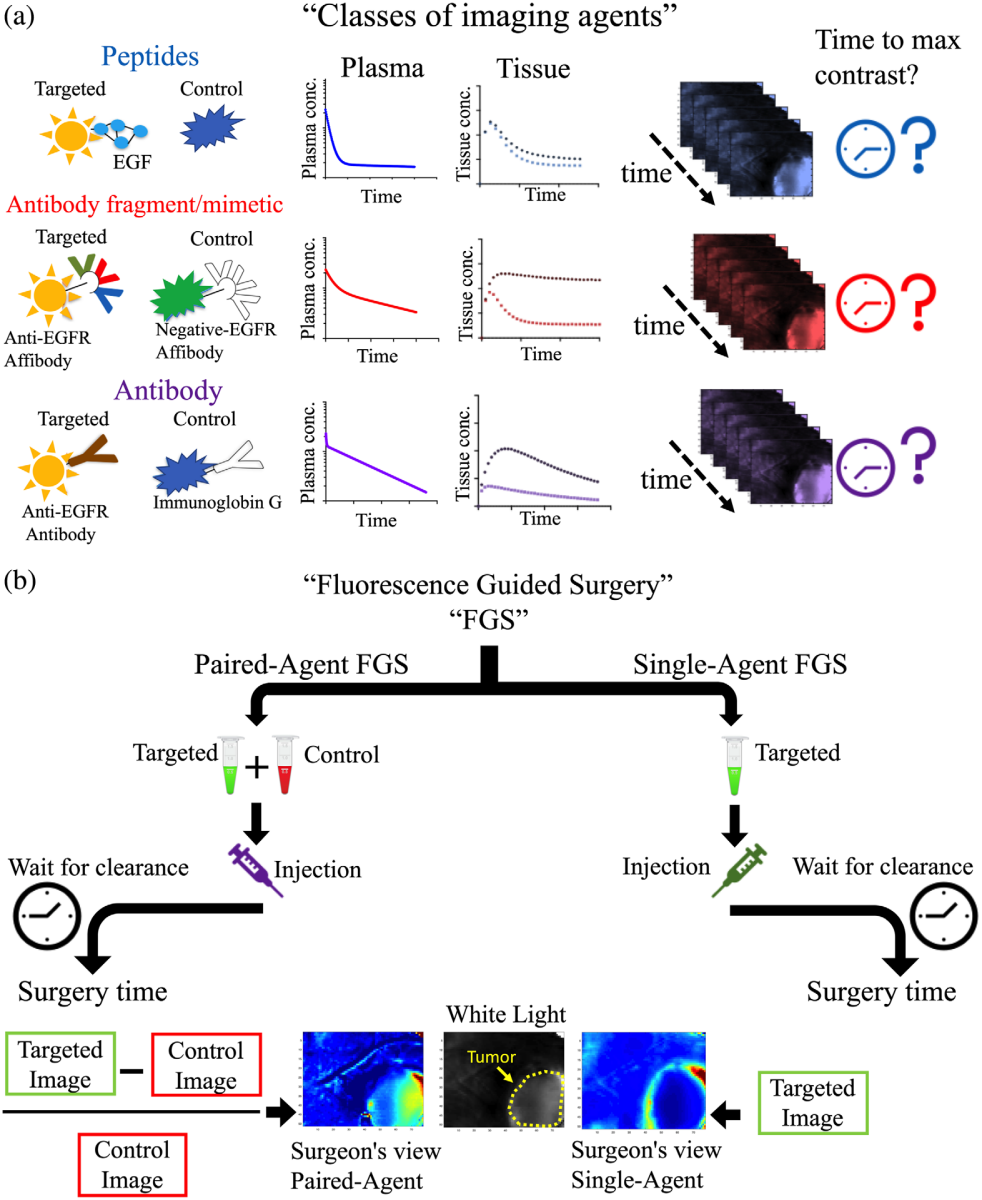
Overview of FGS. (a) Schematic of three classes of protein-based molecular imaging agents that were investigated: peptides, antibody fragment/mimetic, and antibodies (specifically here: targeted: IRDye^®^ 800CW EGF, control: IRDye^®^ 700DX), medium sized antibody fragment/mimetic (targeted: IRDye^®^ 800CW Anti-EGFR affibody, control: IRDye^®^ 680RD negative control affibody^®^), and larger sized antibodies (targeted: IRDye^®^ 800CW Cetuximab, control: IRDye^®^ 700DX IgG). The plasma and tissue clearance and kinetics of these imaging agents differ, and consequently time-to-maximum contrast can differ for each of these groups. The goal of this article was to develop and test a mathematical framework for estimated timing of optimal surgical contrast enabled by different imaging agent properties and different clinically relevant physiological conditions. (b) Two molecular imaging protocols in FGS are discussed: SA and PA imaging. In SA imaging, a targeted imaging agent is injected and there is a delay between injection and the operation to let the unbound imaging agent washout from the body. In PA imaging, a control imaging agent is mixed with the targeted imaging agent and the cocktail is injected. After a delay, the imaging agents can reach an equilibrium level at which improved tumor contrast and a quantitative estimate of target concentrations are achievable, despite residual unbound imaging agents.

To date, the majority of molecular-targeted fluorescent imaging agents employ one of three types of targeting moieties: peptides, antibody fragments or mimetics (e.g., affibodies), or antibodies [Fig. 1(a)].^12^ In general, the imaging agents with smaller targeting moieties (such as peptides and affibodies) exhibit shorter biological half-lives, lower affinities for their targets, and better penetration in the tissue. The fast kinetics of these agents tend to yield optimal tumor-to-normal tissue contrast at <2 to 4 h after agent administration. Conversely, larger antibodies exhibit longer biological half-lives, higher affinities for their targets, and slow/restricted tissue penetration. For these agents, it can take several days following agent administration to reach useful levels of tumor-to-normal tissue contrast. Imaging-agent-specific parameters can be estimated by *in vitro* experiments and all physiological parameters can be estimated from clinically available hemodynamic imaging schemes (e.g., with dynamic contrast enhanced computed tomography^13^ or magnetic resonance imaging^14^ strategies), which could be carried out on a patient-by-patient basis prior to surgery for personalizing FGS dose and imaging/timing protocols. These parameters can be used to generate preoperative patient-specific imaging agent dynamics, thereby personalizing FGS.

This article presents a simple first-order-kinetics mathematical model to predict tumor-to-normal tissue contrast and following injection of a tumor-targeted contrast agent. The contrast is quantified by the contrast-to-variability ratio and the area under the receiver operating characteristic curve (AUROC), parameters that are strongly correlated with the ability of an “ideal observer” to distinguish tumor from normal tissue.^15^ Note that this is not necessarily the case for the often-used metric of “tumor-to-background” used in the fluorescence-guided surgery (FGS) literature (though under shot-noise limited cases it is a good estimate—results not shown). The validity of the mathematical model was tested in human cancer xenograft mouse models using three classes of imaging agents (a native ligand peptide-based agent, an antibody mimetic agent, and an antibody agent) and two distinct imaging techniques [common “single-agent” (SA) methods and “paired-agent” (PA) methods^16^]. While SA imaging is far more common at present, PA molecular imaging strategies [Fig. 1(b)] were also evaluated because they have been shown to minimize the effects of physiological variability by essentially normalizing the signal from a targeted imaging agent to that of a coadministered untargeted (control) imaging agent.^16^ This could allow lower overall agent dosing, more generalizable optimal imaging window selection, and ability to tailor solutions to individual patients, which may support their clinical adoption in the near future as more fluorescent imaging agents see clinical approval.

## 2 Materials and Methods

### 2.1 Theory

This section summarizes the mathematical framework for imaging agent(s) delivery, binding, and washout in tissue, in addition to basic principles of contrast metrics for evaluating “detectability” or ability to “discriminate” tumor from background.

The signal measured from the concentration of the targeted imaging agent in any given region of interest (${\mathrm{ROI}}_{T}$) can be expressed as the weighted sum of the concentration of the targeted imaging agent in the blood plasma ($C_{p,T}$), unbound or “free” in the tissue ($C_{f,T}$), and specifically bound in the tissue ($C_{b,T}$).^17^^,^^18^ It should be noted that the units of $C_{p,T}$ are typically expressed as number of molecules per volume of blood plasma, and $C_{f,T}$ and $C_{b,T}$ are expressed as number of molecules per volume of tissue. For this reason, $C_{p,T}$ is scaled by a fractional blood volume term, $v_{p}$. The control agent was modeled according to the *Kety* model,^19^ which expresses the signal measured from the concentration of the control imaging agent in the same region of interest as the targeted agent, and the concentration of the control imaging agent in any given region of interest (${\mathrm{ROI}}_{C}$) as a weighted sum of the plasma concentration of the control agent ($C_{p,C}$) and its unbound or “free” concentration in the tissue ($C_{f,C}$). The regions-of-interest of the targeted and control imaging agents with respect to time, t, can be represented as follows:

$${\mathrm{ROI}}_{T}(t)=\eta_{T}[\upsilon_{p}C_{p,T}(t)+(1-\upsilon_{p})C_{\text{tissue},T}(t)],$$ (1)

$${\mathrm{ROI}}_{C}(t)=\eta_{C}[\upsilon_{p}C_{p,C}(t)+(1-\upsilon_{p})C_{\text{tissue},C}(t)].$$ (2)

The total tissue concentrations of targeted imaging agent and control imaging agents can be defined as $C_{\text{tissue},T}(t)=C_{f,T}(t)+C_{b,T}(t)$ and $C_{\text{tissue},C}(t)=C_{f,C}(t)$, respectively. The weighting factors, $\eta_{T}$ and $\eta_{C}$, are constants representing the scale between the signal measured from the targeted and control imaging agents and the actual concentration of the agents in the tissue, respectively.

By making the following assumptions that: (1) at least the relative scale of $\eta_{T}/\eta_{C}$ can be determined (perhaps by calibration, or normalization to a “reference tissue” or early time-point imaging,^20^ or estimation by dynamic mathematical models^21^); (2) the plasma input functions, $C_{p,T}(t)$ and $C_{p,C}(t)$, of the targeted and control imaging agents, respectively, are relatively equivalent [i.e., $C_{p}(t)=C_{p,T}(t)=C_{p,C}(t)$], or differences are correctable;^22^ (3) imaging is carried out at a long enough time post-injection that $v_{p}C_{p}(t)\ll C_{f,C}(t)$ and $C_{f,T}(t)$,^23^^–^^25^ and (4) $C_{b,T}\ll \text{the concentration}$ of available targeted biomolecules (“trace” levels),^26^ then the rate of change of the targeted agent concentration in the “free” or interstitial space and in the bound “space” compartments can be represented by the following differential equations:

$$\frac{dC_{f,T}(t)}{dt}=K_{1,T}C_{p}(t)-(k_{2,T}+k_{3})C_{f,T}(t)+k_{4}C_{b,T}(t),$$ (3)

$$\frac{dC_{b,T}(t)}{dt}=k_{3}C_{f,T}(t)-k_{4}C_{b,T}(t),$$ (4)

and the rate of change of the concentration of the control imaging agent in the “free” or interstitial space can be represented as

$$\frac{dC_{f,C}(t)}{dt}=K_{1,C}C_{p}(t)-k_{2,C}C_{f,C}(t),$$ (5)

where $K_{1,T}$ and $K_{1,C}$ represent the first-order rate constants associated with extravasation or leakage of the targeted and control imaging agents, respectively, out of the blood plasma volume and into the extravascular tissue “free”/interstitial space. The first-order rate constants, $k_{2,T}$ and $k_{2,C}$, represent the “efflux” of targeted and control imaging agents, respectively, from the free tissue compartment back to the blood plasma space. Finally, the rate constants, $k_{3,T}$ and $k_{4,T}$, represent the probability of targeted imaging agent binding to and dissociating from its targeted biological molecule (typically a cell-surface protein/receptor), respectively. Since extravasation and efflux parameters are generally dependent on an imaging agent’s size, charge, and lipophilicity,^27^ these parameters were assumed to be the same between the targeted and control imaging agents (i.e., $K_{1}=K_{1,T}=K_{1,C}$; $k_{2}=k_{2,T}=k_{2,C}$, assuming targeted and control agents were selected with similar size, charge, and lipophilicity). We should note that the vascular, tissue, and hemodynamic physiology (specifically blood flow, vascular permeability, and interstitial pressure) in any given region-of-interest will also influence the local $K_{1}$ and $k_{2}$ parameters; however, these factors will have an equivalent influence on both imaging agents as long as the agents are chemically similar.

Taking the difference between Eqs. (1) and (2), dividing that difference by Eq. (2), and substituting the resulting quotient into Eqs. (35) gives

$$\frac{{\mathrm{ROI}}_{T}(t)-{\mathrm{ROI}}_{C}(t)}{{\mathrm{ROI}}_{C}(t)}=\frac{C_{f,T}(t)+C_{b,T}(t)-C_{f,C}(t)}{C_{f,C}(t)}.$$ (6)

Under the assumption that $C_{f,T}$ and $C_{f,C}$ are relatively equivalent, Eq. (6) can be simplified to

$$\frac{{\mathrm{ROI}}_{T}(t)-{\mathrm{ROI}}_{C}(t)}{{\mathrm{ROI}}_{C}(t)}\approx \frac{C_{b,T}(t)}{C_{f,C}(t)}.$$ (7)

Under equilibrium conditions, Eq. (7) is a ratio equivalent to the binding potential, BP: a parameter equal to the product of the targeted imaging agent’s affinity for the targeted receptor (i.e., $K_{A}=1/K_{D}$) and the targeted receptor concentration itself $(B_{\text{avail}})-BP=B_{\text{avail}}\cdot K_{A}$.^28^

### 2.2 Animal Experiments

Human cancer xenograft mouse models were employed as an initial validation of the mathematical framework. All animal procedures were conducted according to protocols approved by the Dartmouth Institutional Animal Care and Use Committee (IACUC). Subcutaneous mouse xenograft models were imaged on epi-illumination fluorescence imaging device. In this study, imaging agents were selected from different “classes” that are used with relatively high frequency in both human and preclinical FGS:

#### 2.2.1 Peptide group

Targeted imaging agent: fluorescently labeled epidermal growth factor (IRDye^®^ 800CW EGF, LI-COR Biosciences, Lincoln, Nebraska); control imaging agent: untargeted fluorescent molecule (IRDye^®^ 700DX, LI-COR Biosciences). Data from this group were sourced from past published work and in-depth details of the experiments can be found in the previous publication.^29^ Briefly, a total of 12 immune-deficient SCID mice (Charles River, Wilmington, Massachusetts) were used. Six mice were implanted subcutaneously on the left flank with $10^{6}$ human glioblastoma cells (U251, ATCC), a cancer cell line known to express moderate levels of the targeted cell-surface receptor, epidermal growth factor receptor (EGFR), and another six mice were implanted with human epidermoid cells (A431, ATCC), known to have high expression of EGFR. Prior to imaging, mice were anesthetized with isoflurane and their tumors were exposed by removing the skin surrounding the tumors. The mice were positioned on an Odyssey Scanner^®^ (LI-COR Biosciences) and 1 nanomole of each of the targeted and control imaging agents were injected intravenously into a tail-vein. Targeted and control agent fluorescence from tumors and surrounding muscle tissue were imaged simultaneously preinjection and then at roughly 2-min intervals post-injection for 1 h. One mouse in A431 group was excluded from the analyses due to insufficient exposed area of muscle in the images (mouse skin is not a good background, as it expresses significant levels of EGFR).

#### 2.2.2 Affibody group

Targeted imaging agent: fluorescently labeled anti-EGFR small antibody-like molecule [IRDye^®^ 800CW (LI-COR) labeled Anti-EGFR affibody^®^, affibody, Solna, Sweden]; control imaging agent: fluorescently labeled negative control small antibody-like molecule [IRDye^®^ 680RD (LI-COR) labeled negative control affibody^®^, affibody]. Data from this group were also sourced from past published work and in-depth details of the experiments can be found in the previous publication.^30^ Briefly, six athymic nude mice were implanted with $10^{6}$ human glioblastoma cells (U251, ATCC) subcutaneously in the right flank in 50  μL of a 1∶1 mixture of Matrigel (BD Biosciences) and complete cell culture media. All tumors were used for imaging and analysis when they reached 100 to $150\;{\mathrm{mm}}^{3}$ in volume. Prior to imaging, mice were administered an i.p. injection of ketamine:xylazine (100∶10  mg/kg) and superficial tissue was removed to expose the tumor and thigh muscle. Each mouse was placed with the tumor and muscle facing down onto a glass side. The mixture of IRDye 800CW-AntiEGFR affibody (0.2 nmol) and IRDye 680RD–control affibody (0.2 nmol) was administered via tail vein injection and scanning was resumed with images taken every minute for the first 15 min and then every 2 to 5 min for a total of 40 min on the Odyssey^®^ Imaging System. One mouse in this group was excluded owing to evidence extravascular injection (i.e., the blood vessel was missed, and the agents were injected into the subcutaneous space).

#### 2.2.3 Antibody group

Targeted imaging agent: fluorescently labeled anti-EGFR antibody (IRDye^®^ 800CW labeled cetuximab); control imaging agent: fluorescently labeled control antibody, immunoglobulin G (IRDye^®^ 700DX labeled mouse IgG). Both agents were labeled based on the fluorophore manufacturer’s (LI-COR Biosciences) specifications. Briefly, three immune-deficient SCID mice (Charles River, Wilmington, Massachusetts) were used. Tumors were implanted subcutaneously on the thigh of the mice with $10^{6}$ human glioblastoma cells (U251, ATCC). Prior to imaging, mice were anesthetized with isoflurane, their tumors were exposed by removing surrounding skin; 1 nanomole each of the targeted and control imaging agents were injected intravenously via a tail vein and targeted and control agent fluorescence from tumors and surrounding muscle tissue were imaged on an Odyssey^®^ Imaging System (LI-COR Biosciences). Scanning was resumed with images taken every minute for the first 15 min and then every 1 h for a total of 10 h (the longest time point that the IACUC protocol allowed).

### 2.3 Image Quality Metrics

The ultimate goal of molecular FGS is to improve surgeons’ abilities to distinguish between different tissue types (e.g., tumor and normal) during an operation. In this study, two metrics were used to compare the ability of the SA and PA imaging: (1) AUROC and contrast-to-variability ratio (CVR). AUROC is a commonly used metric used to quantify how well an “ideal observer” would be able to discriminate two distinct groups with their own means and variances (a maximum AUROC value of 1 would represent that 100% sensitivity and 100% specificity are possible). However, AUROC requires knowledge of the true status of each pixel to evaluate the performance of an imaging modality. On the other hand, CVR is a metric that correlates strongly with AUROC and can estimate the performance of imaging based on estimated contrast between the two selected regions:

$$\mathrm{CVR}=\frac{\left|\mu_{T}-\mu_{B}\right|}{\sqrt{\sigma_{T}^{2}+\sigma_{B}^{2}}},$$ (8)

where $\mu_{T}$ and $\mu_{B}$ represent the mean signals measured in tumor and normal tissue surrounding the tumor, respectively, and $\sigma_{T}$ and $\sigma_{B}$ represent the respective standard deviations in these mean signals, which has been shown to correlate well with the “ideal-observer” ability to perform a discrimination task.^31^ The dynamics of AUROC and CVR after imaging agent injection were plotted for mean±SD in each group.

### 2.4 Data Analyses

Image frames of the targeted and control imaging agents were analyzed in MATLAB (MathWorks^®^). Corresponding preinjection images were subtracted from all post-injection imaging frames of the targeted and control imaging agents to remove effects of autofluorescence. The two fluorescence channels in each experiment were normalized using an early time-point pixel-by-pixel normalization method^20^ to correct for differences in signal intensities between each pair of the imaging agents. In addition, a deconvolution technique^22^ was applied to correct for measured differences in the plasma kinetics of the targeted and control agents. Temporal kinetics of the imaging agents in the tumor and normal tissue were plotted. $BP_{\text{ratio}}$ was estimated in muscle and tumor using Eq. (6) and averaged over the selected regions-of-interest.

### 2.5 Simulations

To simulate dynamic (temporal) concentration and signal-curves for targeted and control imaging agents, differential equations in Eqs. (35) were solved by numerical methods. These methods required input values for all parameters, $K_{1,T},k_{2,T},k_{3,T},k_{4,T},K_{1,C}$, and $k_{2,C}$, as well as a plasma input function, $C_{p}(t)$. To mimic the analogous simulated data to each of the mouse in the experiments, we first fitted the Kety model [the solution of one-compartment model in Eq. (5)] to the uptake of untargeted imaging agent in each mouse using population-based plasma input functions from our previous publications.^25^^,^^32^ A biexponential function was used to represent the pharmacokinetics of the imaging agents in the body. Plasma kinetic parameters of A, B, α, and β with the form of $C_{P}(t)=Ae^{-\alpha t}+Be^{-\beta t}$ for EGF were fitted with the MATLAB lsqcurvefit.m function, and for affibody and antibody were obtained from the previous study.^32^ This formed the plasma input function required to fit the untargeted data to one-compartment model (Table 1). After fitting the model and finding the map of $K_{1,C}$ and $k_{2,C}$ in the tumor and normal regions for each mouse, we found the mean±SD of these values. We used the mean of these values across the mice in each group as the input to our model. The *in vivo* specific binding rate constant was assumed to be equal to the product of the concentration of targeted biomolecules available for binding, $B_{\text{avail}}$, and the “*in vitro* on-rate constant,” $k_{\text{on}}$ for “trace” level imaging.^28^ The *in vitro* $k_{\text{on}}$ rate constant can vary greatly depending on the characteristics of the imaging agent/biomolecule pair and can reach levels of $10^{6}\;M^{-1}.\min^{-1}$; whereas $B_{\text{avail}}$ is not typically higher than about $10^{-6}\;M$. This means that the physiological range of $k_{3}$ is generally between 0 and $1\;\min^{-1}$. Similar to $k_{\text{on}}$, $k_{\text{off}}$ can vary greatly by many orders of magnitude but generally scales with $k_{\text{on}}$. At least for reversible binding agents, “stickier” agents tend to require more complicated multivalent binding sites that make the probability of binding, $k_{\text{on}}$, lower since the probability that a complex binding site meets the targeted receptor in the exact correct orientation goes down with complexity. In general, $k_{\text{off}}$, and therefore $k_{4}$, is measured between 0 and $1\;\min^{-1}$, similar to $k_{3}$. The *in vivo* dissociation rate constant is typically assumed to be directly equal to $k_{\text{off}}$, the *in vitro* off-rate constant. The list of typical $k_{\text{on}}$ and $k_{\text{off}}$ parameters for each imaging agent is shown in Table 1. The final step in simulating the uptake curves was to convert the experimental equilibrium BPs to $B_{\text{avail}}s$ ($BP=B_{\text{avail}}/K_{D}$) and use them as the inputs for the concentration of the targeted biomolecules available for binding.

**Table 1:** Description of parameters used in the simulation study.^17^^,^^26^^,^^32^

Symbol (unit)	Description	Peptide	Affibody	Antibody
A (unitless)	Distribution fraction	0.02	0.6	0.4
α ($\min^{-1}$)	Distribution phase constant	0.3	0.2	0.03
B (unitless)	Elimination fraction	2E-03	0.4	0.5
β ($\min^{-1}$)	Elimination phase constant	6E-04	5E-03	3E-04
Tumor $K_{1}$^a^ ($\min^{-1}$)	Extravasation rate from plasma to tumor tissue	0.4±0.2	0.01±0.04	2E-04
Tumor $k_{2}$^a^ ($\min^{-1}$)	Efflux rate from tumor tissue to plasma	0.2±0.2	0.3±0.6	8E-03
Normal $K_{1}$^a^ ($\min^{-1}$)	Extravasation rate from plasma to normal tissue	0.1±0.03	0.01±0.02	2E-04
Normal $k_{2}$^a^ ($\min^{-1}$)	Efflux rate from normal tissue to plasma	0.02±0.01	0.3±0.2	8E-03
$k_{\text{on}}$ (${\mathrm{nM}}^{-1}.\min^{-1}$)	Binding rate	0.1	0.04	0.16
$k_{\text{off}}$ ($\min^{-1}$)	Dissociation rate	0.1	0.1	0.07
$K_{D}$ (nM)	Equilibrium dissociation rate constant	1	3	0.4

a For peptide and affibody groups, these values are estimated from the experimental part of this study for U251 tumors. For antibody group, the average values are taken from the references. Data show within group variance.

The system of differential Eqs. (35) was solved with the MATLAB ode45.m function, which uses a fourth-order, five-step Runge–Kutta method, and adaptive quadrature to generate the curves for targeted and control imaging agents in tumor and muscle at optimal time points (e.g., higher density where rates of change are higher—“adaptive quadrature”) within a provided timespan. Data were then interpolated to the desired timing frequency. We assumed that at time zero (t=0), the concentration of all imaging agents was zero in the regions-of-interest. Table 1 displays a summary of the pharmacokinetic and physiological values that were selected for each class of imaging agent molecules in this study. To mimic the biological variabilities, we assumed that in the tumor and normal regions, the map of $K_{1}$ and $k_{2}$ values (assuming both targeted and control imaging agents follow the same kinetics) would exhibit similar mean and variance to the experimental data. For A431-peptide group the mean±SD of $K_{1}$ and $k_{2}$ are 0.3±0.2 and 0.2±0.2 in the tumor and 0.1±0.04 and 0.03±0.01 in the muscle, respectively. After generating the uptake of the targeted and control imaging agents in tumor and surrounding tissue, we then converted these curves to 16-bit dynamic-range (assuming maximum signal in each case was 20% of the full-dynamic range of a 16-bit shot-noise limited detector—adding noise with the MATLAB poissrnd.m function). It should be noted that second-order or saturation binding was not included; however, we always maintained bound concentrations that were less than 5% of $B_{\text{avail}}$ (i.e., “trace” conditions). More complex models could be adapted for high dose imaging agents if required.^26^

### 2.6 Analytical Approximation of Time-to-Maximum CVR for Single-Agent and Paired-Agent Imaging

The differential equations governing SA and PA imaging agent kinetics [Eqs. (35)] were further simplified to approximate an expression for CVR (contrast between tumor and normal tissue) by assuming that the plasma input function could be approximated by a monoexponential decay function (see the Supplementary Materials). The simplified equation suggested that the time-to-maximum CVR for SA imaging depends on $K_{1}$ and $k_{2}$ of the targeted imaging agent in the tumor and normal tissue and the BP:

$$T_{\max,\mathrm{SA}}≅\frac{\ln(\frac{K_{1}^{\prime }}{K_{1}})}{k_{2}^{\prime }-k_{2a}},$$ (9)

where $T_{\max,SA}$ is the time of the maximum CVR for SA imaging. $K_{1}$ and $K_{1}^{\prime }$ are the extravasation rate constants from the blood to the tumor and normal tissue, respectively. $k_{2}^{\prime }$ is the efflux rate constant from the normal tissue to the blood plasma. The “apparent efflux rate” from the tumor to the blood is represented as $k_{2a}=k_{2}/(1+BP)$,^33^ where $k_{2}$ represents the efflux rate constant in the tumor. The analytical solution for PA imaging depends on the efflux rate constant in the tumor tissue, as well as the BP:

$$T_{\max,PA}≅\frac{10}{k_{2}-k_{2a}}[\ln(\frac{1}{k_{2}^{2}}-\frac{1}{k_{2a}^{2}})-\ln(\frac{2}{k_{2}^{2}}-\frac{2}{k_{2}k_{2a}})].$$ (10)

For details on the derivations of Eqs. (9) and (10), see the Supplementary Materials. To test the correlation between the estimations of the CVR $T_{\max}$ using Eqs. (9) and (10) and the true time of max contrast in both SA and PA protocols, the uptake and binding properties of hypothetical imaging agents—representing four classes of imaging agents: peptides, low molecular weight antibody fragments, high molecular weight antibody fragments, and antibodies—were simulated over a range of biological properties (Table S1 in the Supplementary Materials and Table 2). Typical kinetic parameter ranges of these classes of imaging agents were estimated from the literature by converting the permeability of these molecules to $K_{1}$ and $k_{2}$, and by finding BP from $B_{\text{avail}}$ and $K_{D}$ values (Table 2).^34^ Parametric ranges were sampled in 1000 iterations where each iteration involved randomly selecting a value from each defined range (using *rand()* in MATLAB; Table 2).

**Table 2:** Description of parameters used in the simulation study based on human cancer parameters. The numbers in each cell indicate the minimum and maximum (range) of their corresponding variables used in the simulations.

Name	Examples	Tumor $K_{1}$ ($\min^{-1}$)	Tumor $k_{2}$ ($\min^{-1}$)	Normal $K_{1}$ ($\min^{-1}$)	Normal $k_{2}$ ($\min^{-1}$)	BP
Peptides	Linear, Cyclin	[0.18, 0.63]	[0.29, 1.03]	[0.16, 0.43]	[0.26, 0.7]	[13.5, 67.5]
Low MW antibody-fragments	Centyrin, Affibody, Knottin	[0.0198, 0.0952]	[0.032, 0.156]	[0.0196, 0.0906]	[0.032, 0.148]	[13.12, 15]
High MW antibody-fragments	Fab, Diabody, scFv	[0.002, 0.01]	[3.28e-3, 0.0164]	[0.002, 0.01]	[0.003, 0.016]	[0.187, 3.75]
Antibodies	IgG, Minibody	[2.1E-04, 5.6E-03]	[2.4E-04, 9.1E-03]	[3.1E-04, 5.6E-03]	[5.1E-04, 9.2E-03]	[0.0185, 0.037]

### 2.7 Statistics

All statistical analyses were carried out in Graphpad Prism 8. Linear regression was employed to compare the simulation results with the experimental data for AUROC and CVR at their maximum values for SA and PA imaging. Statistical significance was based on p<0.05. Pearson correlation was used to correlate the experimental and simulation results. All data were presented as mean±SD.

## 3 Results

### 3.1 Animal Experiments

**Fig. 2:**
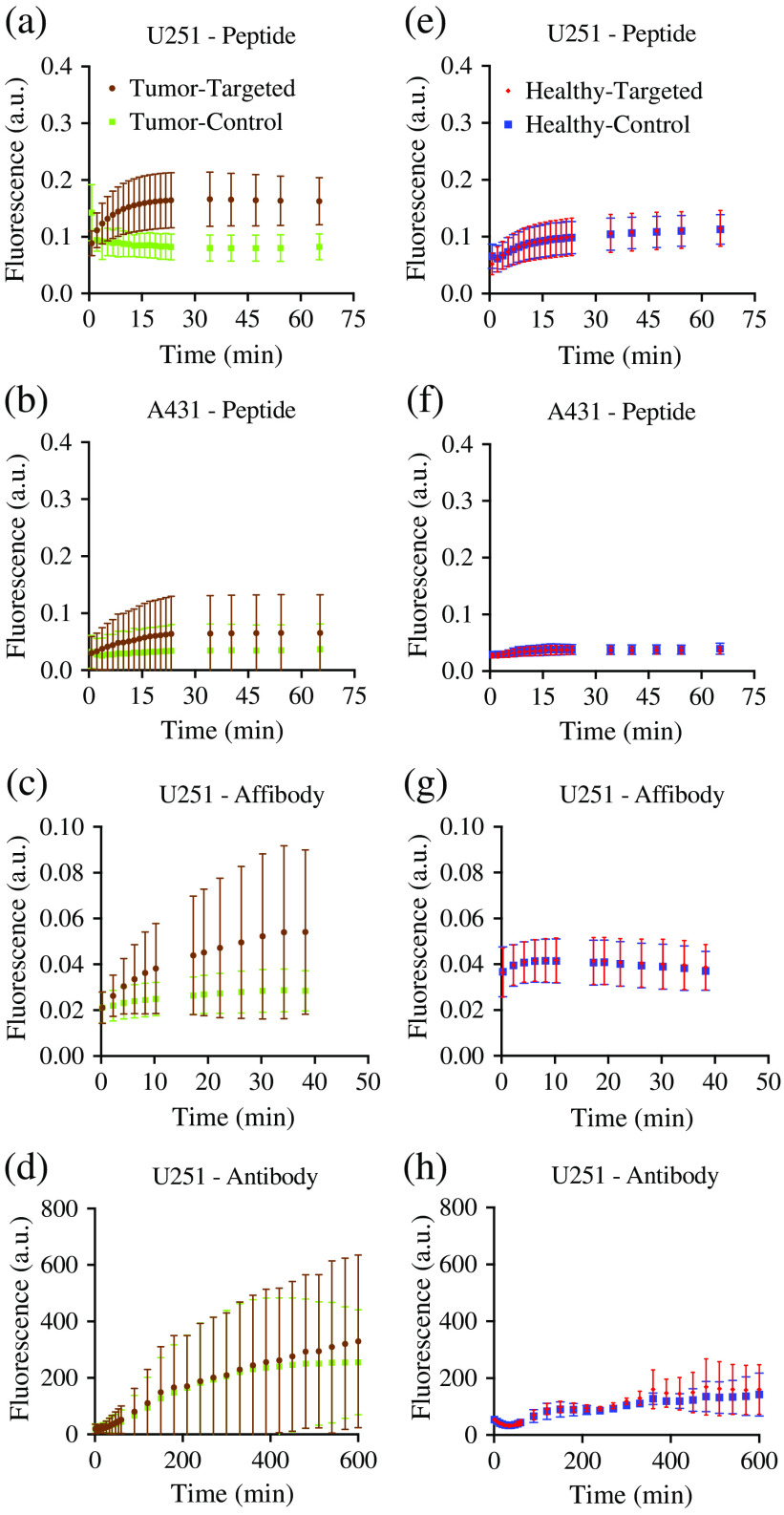
Targeted and control imaging agents in three different imaging agent classes: peptides, affibodies, and antibodies. Mouse human cancer xenograft (subcutaneous thigh tumor models) fluorescent imaging dynamics from the peptide-based imaging agent (targeted: IRDye^®^ 800CW EGF, control: IRDye^®^ 700DX) studies are presented from the moderate EGFR-expressing cell line (human glioblastoma; U251); the high EGFR-expressing cell line (human epidermoid, A431); the affibody-based imaging agent study in U251 xenografts (targeted: IRDye^®^ 800CW anti-EGFR affibody, control: IRDye^®^ 680RD negative control affibody^®^); the antibody-based imaging agent study in U251 xenografts (targeted: IRDye^®^ 800CW Cetuximab, control: IRDye^®^ 700DX IgG). (a)–(d) The mean fluorescence measured for the targeted and control imaging agents in the tumors in each group (errors are SD between animals). (e)–(h) The same information in a proportionally sized region-of-interest in the muscle surrounding the tumors in each case.

The average fluorescence signals in the tumor and muscle for all imaging agents under study (peptide-, affibody-, and antibody-based targeted and control agents) are shown in Fig. 2. The signal of targeted and control imaging agents in the tumor [Figs. 2(a)–2(d)] and muscle (normal tissue) [Figs. 2(e)–2(h)] regions were averaged over their respective regions-of-interest for each mouse. All peptide and affibody molecules, regardless of tumor type, achieved a relatively steady state 15 to 40 min after injection. Due to animal care requirements, continuous imaging of antibodies was only performed out to 10 h and was not observed to have reached an equilibrium [Figs. 2(d) and 2(h)].

**Fig. 3:**
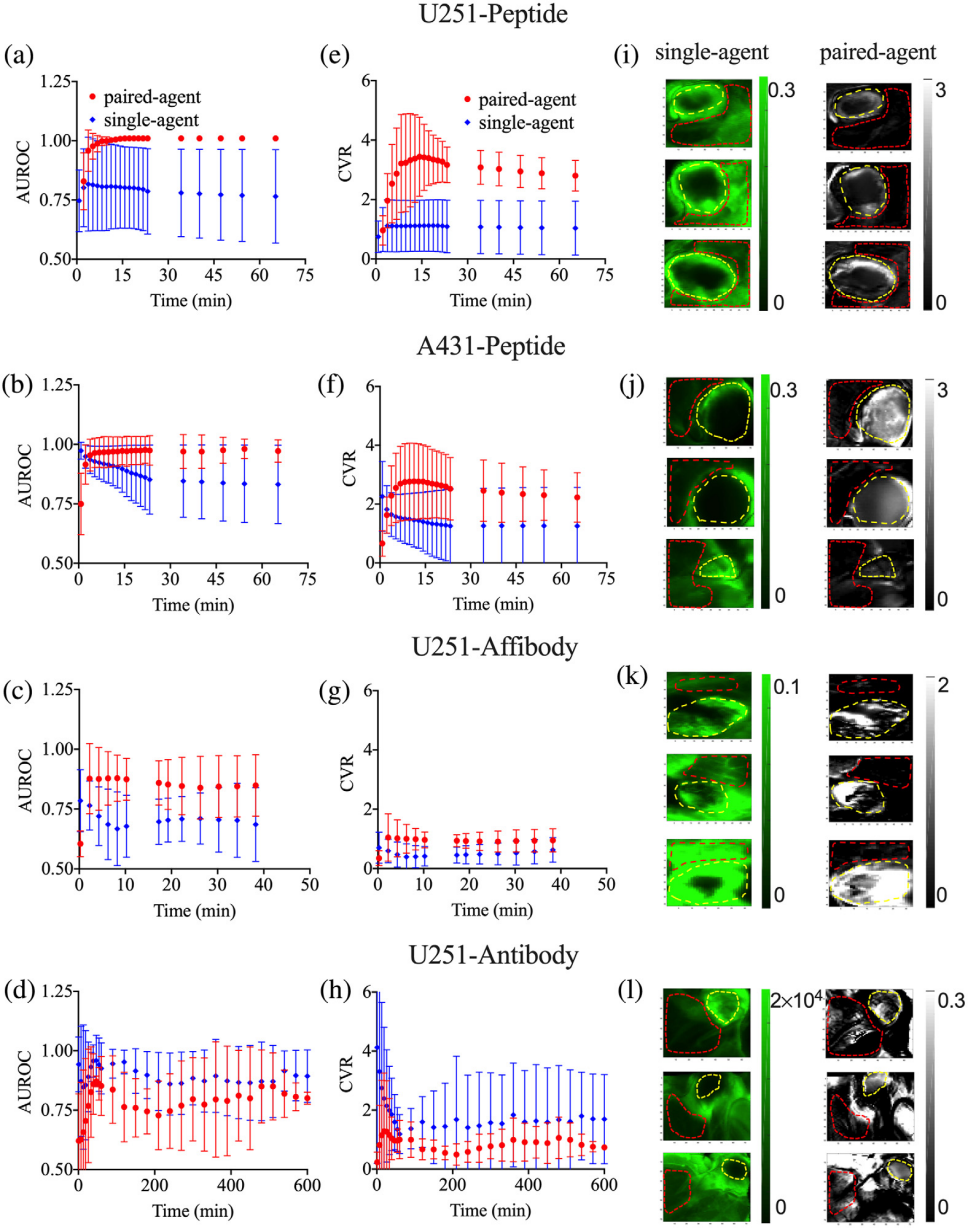
Comparison of the PA and SA FGS protocols. The AUROCs and the CVRs measured from PA (red data) and SA (blue data) analyses of the experimental results as a function of time post-imaging-agent injection. The mean of the AUROCs measured in each group (errors are SD between animals) for (a) the U251-peptide group, (b) the A431-peptide group, (c) the U251-affibody group, and (d) the U251-antibody group. The CVR measured in each group (errors are SD between animals) for (e) the U251-peptide group, (f) the A431-peptide group, (g) the U251-affibody group, and (h) the U251-antibody group. The third column depicts maps of targeted imaging agent fluorescence the SA (targeted; green) and the PA binding potentials ($BP_{\text{ratio}}$; grayscale) for three randomly selected mice in each of (i) the U251-peptide group, (j) the A431-peptide group, (k) the U251-affibody group, and (l) the U251-antibody group. The yellow dashed circles depict the location of the tumors and the red dashed circles show the normal muscle tissue surrounding the tumor that were selected as representative of “background.” The ROIs were selected based on the white-light images (not shown).

Temporal changes in the AUROC for each group of mice in SA and PA imaging are shown in Figs. 3(a)–3(d). The mean±SD of AUROCs at their maximum values in each animal from the U251-peptide group were 0.9±0.2 and 1.0±0.0 (all were 1) for SA and PA, respectively. For the A431-peptide group, these means were 0.97±0.03 and 0.98±0.04 for SA and PA, respectively. The mean AUROCs of the U251-affibody group were 0.84±0.05 and 0.94±0.06 in the SA and PA groups, respectively. In the U251-antibody group, these means were 0.9±0.1 and 0.80±0.02, respectively. Temporal changes in CVR [see Eq. (8)] for each group of mice in SA and PA imaging are shown in Figs. 3(e)–3(h). The mean±SD of CVRs at the maximum value in each animal from the U251-peptide group were 1.3±0.8 and 4±1 for SA and PA, respectively. On the other hand, for the A431-peptide group, these means were 2±1 and 3±1 for SA and PA, respectively. The mean CVRs of the U251-affibody group were 2±1 and 1.6±0.5 in the SA and PA groups, respectively. In the U251-antibody group, these means were 2±2 and 0.73±0.07, respectively. We should note here that one of the three mice in the antibody group exhibited a tumor uptake that was five times greater than the other two mice in this group; as a result, the max CVR of SA imaging for antibody was around four times higher than PA (these variabilities are corrected for by the commensurate increase in control agent signal in the PA analysis). When we excluded the mouse with higher uptake from the analysis, mean maximum CVRs of SA and PA in the antibody U251 group were 0.8±0.3 and 0.71±0.08, respectively. Between 1 and 40 min were the approximated times post-agent-injection to reach the maximum contrast for the U251-peptide, A431-peptide, and U251-affibody groups. After the imaging agents pass their peaks, they stay at equilibrium for longer time windows. Note: imaging agent signals in all experiments were greater than 10 times the autofluorescence background in all tissue for all time points measured.

### 3.2 Simulations

**Fig. 4:**
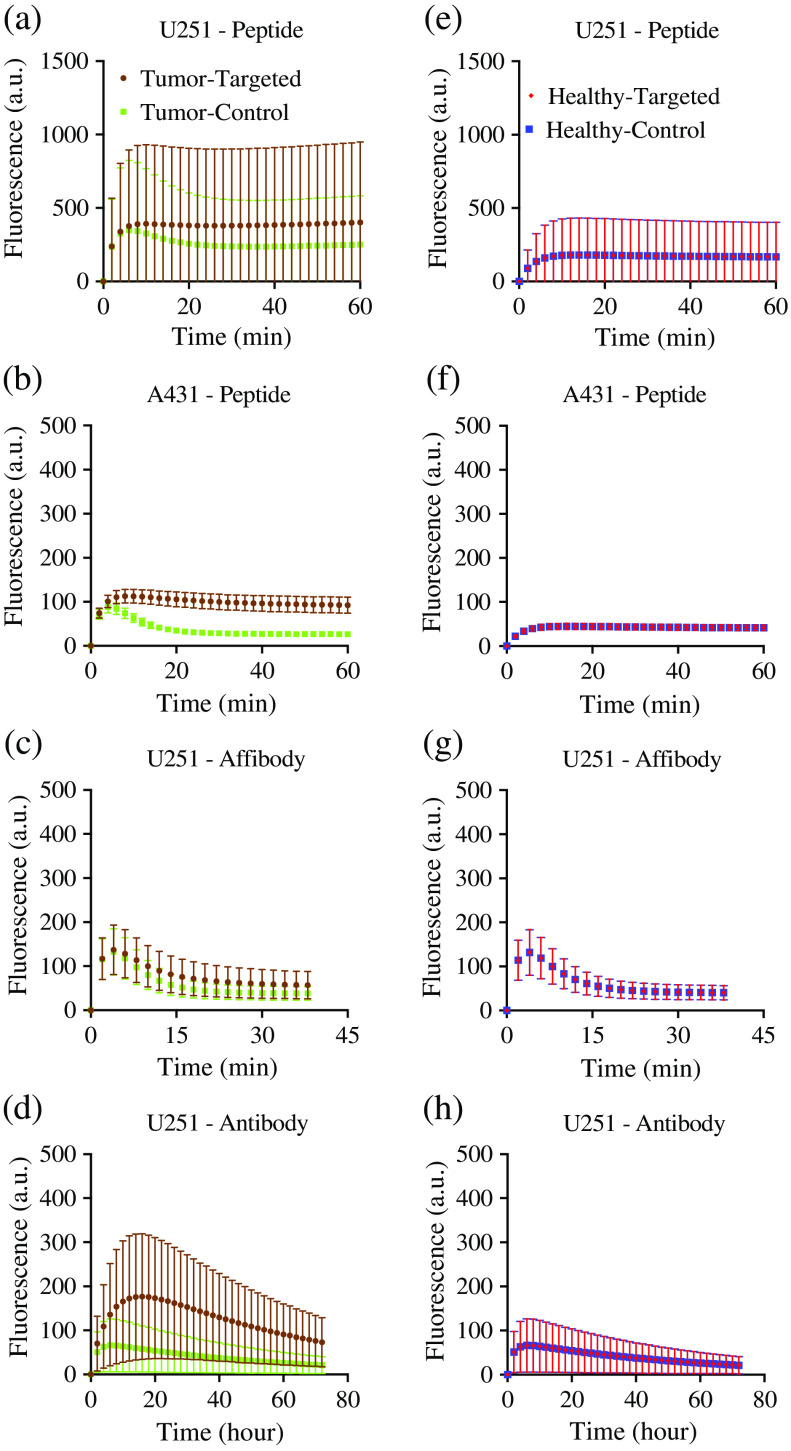
Simulation results for three different classes of imaging agents: peptides, affibodies and antibodies. Simulated fluorescence signal are presented for mouse human cancer xenograft (subcutaneous thigh tumor models) fluorescent imaging dynamics from the peptide-based imaging agent (targeted: IRDye^®^ 800CW EGF, control: IRDye^®^ 700DX) from the moderate EGFR-expressing cell line (human glioblastoma; U251); the high EGFR-expressing cell line (human epidermoid, A431); the affibody-based imaging agent study in U251 xenografts (targeted: IRDye^®^ 800CW anti-EGFR affibody, control: IRDye^®^ 680RD negative control affibody^®^); and the antibody-based imaging agent study in U251 xenografts (targeted: IRDye^®^ 800CW Cetuximab, control: IRDye^®^ 700DX IgG). (a)–(d) The mean fluorescence measured for the targeted and control imaging agents in the tumors in each group (errors are SD between animals). (e)–(h) The same information in a proportionally sized region-of-interest in the muscle surrounding the tumors in each case.

Results of the targeted and control agent curves in the tumor and muscle for each simulation group are presented in Fig. 4 (each simulation group was developed to mimic the conditions for the U251 and A431 tumor versus muscle uptake of the peptide-, affibody-, and antibody-based experimental groups).

**Fig. 5:**
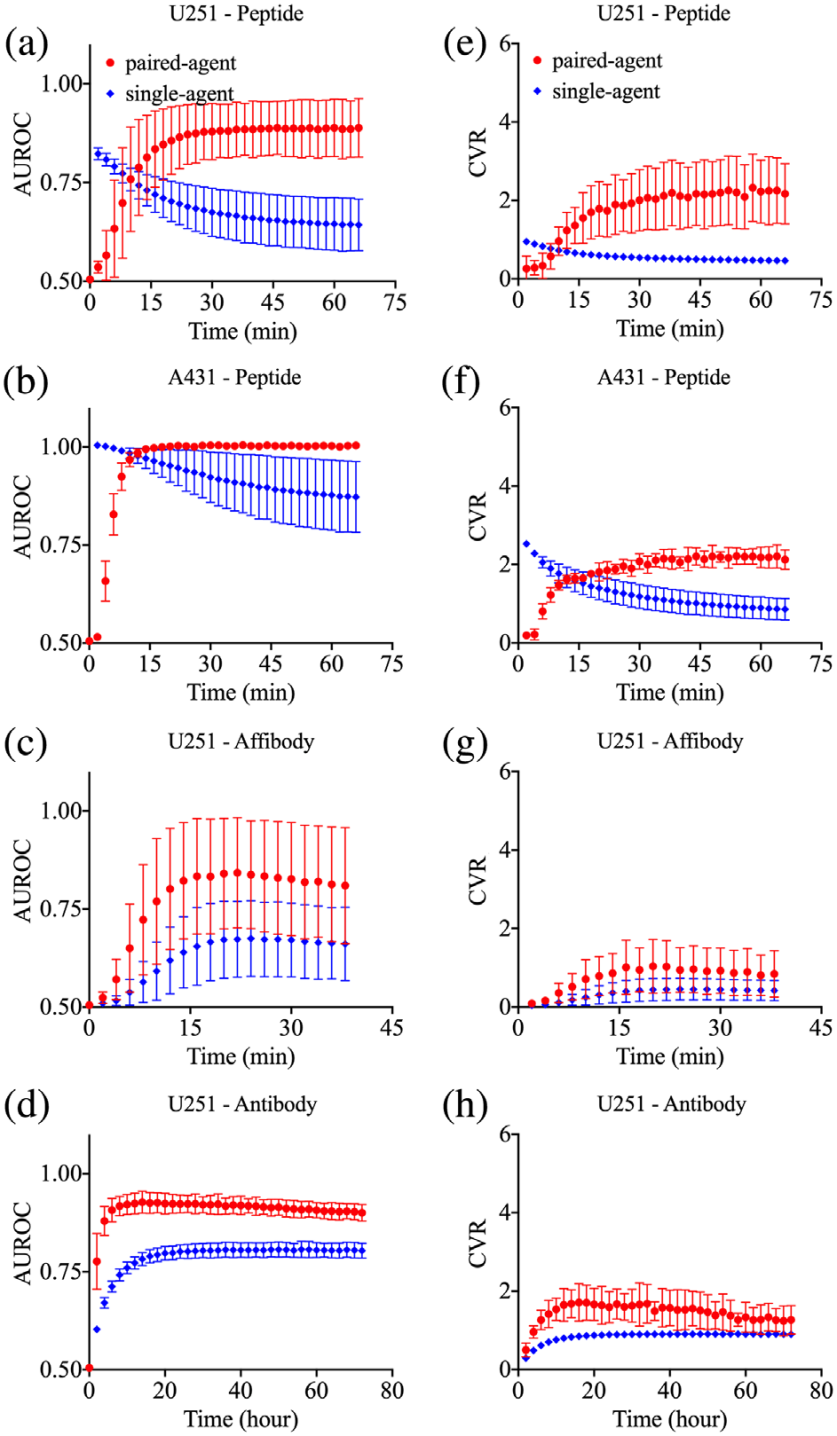
Simulation results for three different classes of imaging agents: peptides, affibodies and antibodies. The mean±SD of AUROC (blue = SA, red = PA) for (a) the U251-peptide, (b) the A431-peptide, (c) the U251-affibody and (d) the U251-antibody simulated groups. The mean±SD of CVR (blue = SA, red = PA) for (e) the U251-peptide, (f) the A431-peptide, (g) the U251-affibody, and (h) the U251-antibody simulated groups.

For the particular parameter ranges simulated (Table 1), Fig. 5 presents the AUROCs and CVRs for SA and PA approaches over time. Temporal changes in AUROC for each simulated group of mice in SA and PA imaging are shown in Figs. 5(a)–5(d). The mean±SD of AUROCs at the maximum value in each animal from the peptide U251 group were 0.82±0.01 and 0.89±0.06 for SA and PA, respectively. For the peptide A431 group, these means were 1±0 (all 1 s) and 1±0 (all 1 s) for SA and PA, respectively. The mean AUROCs of the affibody U251 group were 0.67±0.09 and 0.8±0.1 in the SA and PA groups, respectively. In the antibody U251 group, these means were 0.80±0.02 and 0.90±0.02, respectively. Temporal changes in CVR for each simulated group of mice in SA and PA imaging are shown in Figs. 5(e)–5(h). The mean±SD of CVRs at the maximum value in each animal from the peptide U251 group were 0.95±0.04 and 2.4±0.9 for SA and PA, respectively. On the other hand, for the peptide A431group, these means were 2.52±0.09 and 2.3±0.2 for SA and PA, respectively. The mean CVRs of the affibody U251 group were 0.5±0.3 and 1.1±0.7 in the SA and PA groups, respectively. In the antibody U251 group, these means were 0.90±0.07 and 1.3±0.4, respectively.

**Fig. 6:**
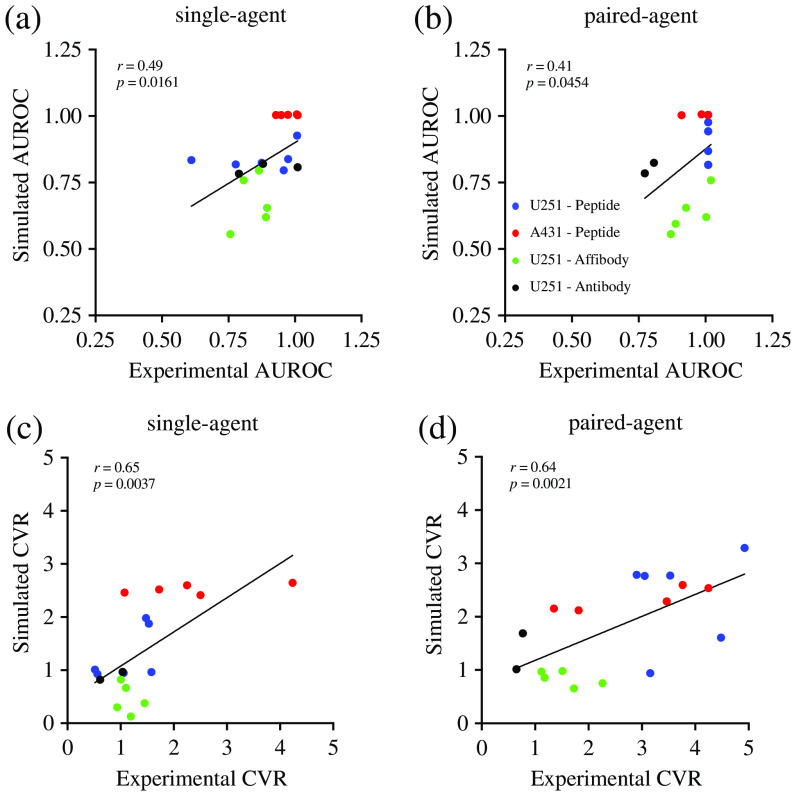
Comparison between experimental and estimated simulation results. Correlations between estimated and simulated areas under the receiver operating characteristic curve (AUROCs) for (a) SA and (b) PA imaging protocols. CVRs for (c) SA and (d) PA imaging agent protocols. The correlations are tested at the frame of maximum value.

We evaluated the ability of the model to describe and potentially predict imaging agent behavior *in vivo* given basic physiological parameters. We correlated experimental and simulation results of SA and PA AUROC at the maximum values [Figs. 6(a) and 6(b)]. Experimental and simulation results of AUROC showed statistically significant correlations for both SA (Pearson r=0.49, p<0.05) and PA (Pearson r=0.41, p<0.05). The linear regression lines were Y=0.61X+0.28 [Fig. 6(a)] and Y=0.81X+0.06 [Fig. 6(b)] for SA and PA, respectively. Mean experiment-based AUROC values for the SA measurements [Fig. 6(b)] were significantly higher (p<0.01) compared to values for the SA measurements [Fig. 6(a)]: 0.9±0.2 versus 0.7±0.2, respectively. Experimental and simulation results of SA and PA CVR at the maximum value [Figs. 6(c) and 6(d)] showed statistically significant correlation, (Pearson r=0.65, p<0.01) and (Pearson r=0.64, p<0.01) for SA and PA, respectively. The linear regression lines were Y=0.64X+0.43 [Fig. 6(c)] and Y=0.41X+0.76 [Fig. 6(d)] for SA and PA, respectively.

**Fig. 7:**
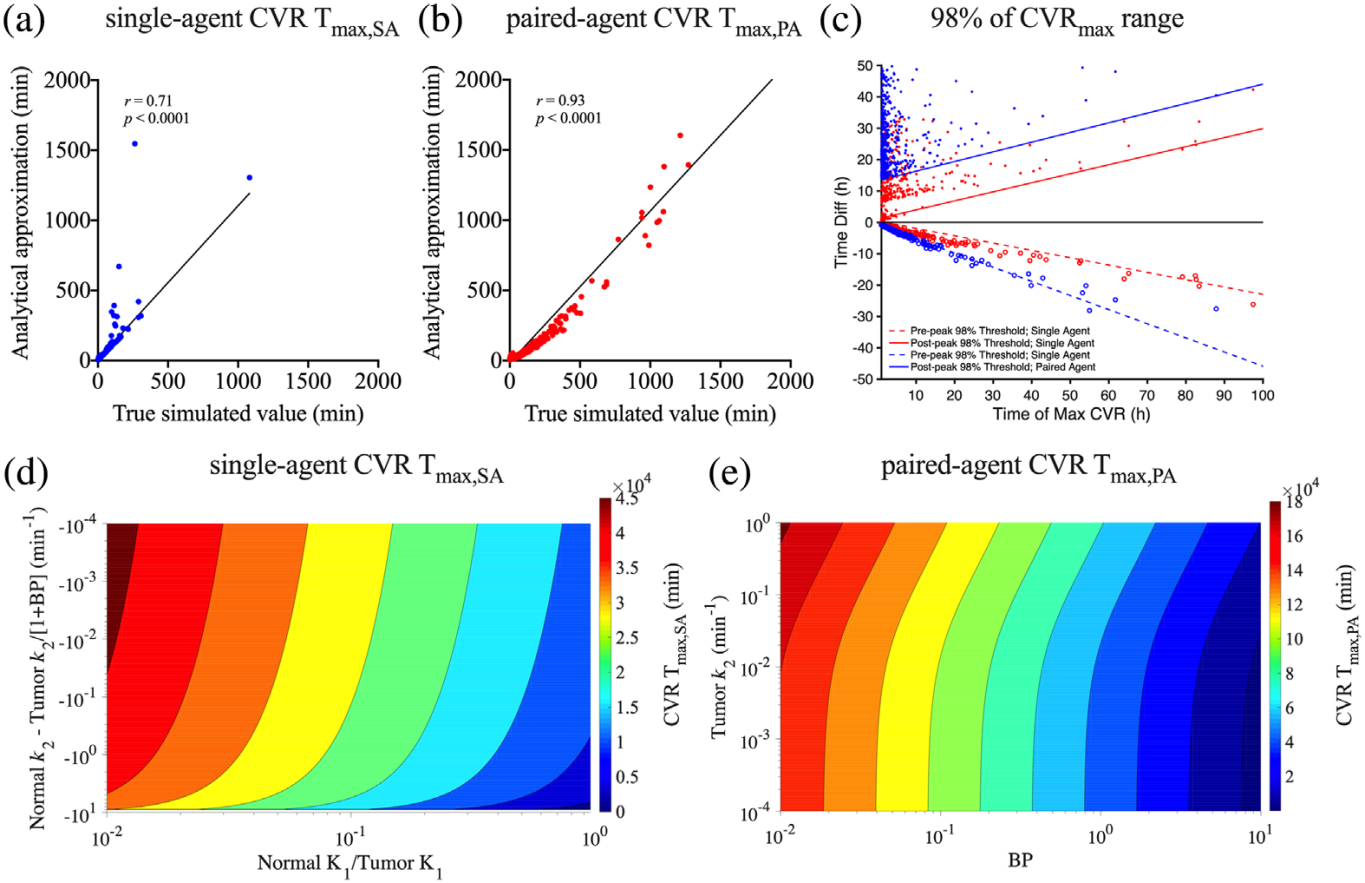
Predicting time-to-maximum tumor contrast (CVR $T_{\max}$) after imaging agent injection based on human cancer parameters. Correlations between true simulated values and estimations by the analytical solutions of CVR $T_{\max}$ for (a) SA and (b) PA imaging techniques. (c) Correlations between true simulated values of CVR $T_{\max}$ and estimations at 98% CVR before and after CVR $T_{\max}$ for SA and PA imaging techniques. Time Diff refers to the difference in time between the pre 98% of maximum CVR threshold pre-$T_{\max}$ (circle data) or post-$T_{\max}$ (solid dots) and the CVR $T_{\max}$. Red data refer to the SA simulations and blue data to the PA simulations. The input parameters were perturbed to cover a range of parameters from small peptides to large antibodies with different binding kinetics. (d) Predicted CVR $T_{\max,\mathrm{SA}}$ based on blood extravasation rate constants of the targeted imaging agent in the tumor and “normal”/background tissues, respectively ($K_{1}$), the corresponding tissue-to-blood efflux rate constants ($k_{2}$), and the tumor binding potential (BP). (e) Predicted CVR $T_{\max,\mathrm{PA}}$ based on tissue-to-blood efflux rate constant in the tumor ($k_{2}$) and the tumor binding potential (BP).

Based on our simplified analytical approximations for the time-to-maximum CVR ($T_{\max}$) [Eqs. (9) and (10); see the Supplementary Materials], CVR $T_{\max,\mathrm{SA}}$ for SA FGS was dominated by the extravasation and efflux rates ($K_{1}$ and $k_{2}$) of the imaging agent in both tumor and normal tissues, and the BP (which itself depends on $k_{\text{on}}$, $k_{\text{off}}$, and $B_{\text{avail}}$) of the tumor. On the other hand, CVR $T_{\max,\mathrm{PA}}$ for PA imaging was dominated by the $k_{2}$ of the tumor and the BP. True simulated $T_{\max}$ values and the estimations by analytical approximations of SA and PA CVR [Figs. 7(a) and 7(b)] showed statistically significant correlations: r=0.71 (p<0.0001), and r=0.93 (p<0.0001) for SA and PA, respectively. The linear regression lines were Y=1.09X−6.88 [Fig. 7(a)] and Y=1.07X−14.26 [Fig. 7(b)] for SA and PA CVR $T_{\max}$, respectively. Note: a number of approximations were made to achieve expressions in Eqs. (9) and (10), and while the correlations between both equations estimate of CVR $T_{\max}$ and actual CVR $T_{\max}$ were strong [Figs. 7(a) and (b)], Eq. (10) needed to be scaled by a factor of 10 to correct for a proportionality bias compared to the raw derived Eq. (S15) of the Supplementary Materials.

To find an acceptable time window for surgery during which the tumor contrast is still high, we calculated the times at 98% ${\mathrm{CVR}}_{\max}$ before and after CVR $T_{\max}$ [Fig. 7(c)]. The average PA imaging range about 98% of the CVR $T_{\max}$ began at least two times earlier than that for SA and remained high for at least two times longer after the CVR $T_{\max}$.

Rough mapping of predicted CVR $T_{\max,\mathrm{SA}}$ and $T_{\max,\mathrm{PA}}$ over ranges of $K_{1}^{\prime }/K_{1}$ versus $k_{2a}$ (for SA), and BP and $k_{2}$ (for PA) is presented in Figs. 7(d) and 7(e), respectively. These mappings can provide order-of-magnitude estimates, essentially as a look-up table, if the parameters on the access can be approximated from preoperative DCE imaging for individual patients. For more precise estimates, Eqs. (9) and/or (10) can be used.

The time-to-maximum contrast for different classes of imaging agents was estimated using the input parameters from a combination of sources^34^^–^^36^ (see Table S1 in the Supplementary Materials) for a tumor with target overexpression of 150 nM (i.e., $B_{\text{avail}}=150\;\mathrm{nM}$). The permeability parameters were converted to $K_{1}$ and $k_{2}$ rate constants using an average surface area value (Table 2). Table 2 displays the summary of the input parameters that were used to predict the CVR $T_{\max}$ values, and Table 3 lists the estimated values of CVR $T_{\max}$ for different classes of imaging agents. All the $T_{\max}$ estimations were consistent with experimental observations in this study and from similar studies (see Table 1 and the Supplementary Materials).

**Table 3:** Time of the maximum CVR ($T_{\max}$) after intravenous injection of different imaging agents based on human cancer parameters.

Name	CVR $T_{\max,\mathrm{SA}}$	CVR $T_{\max,\mathrm{PA}}$
Peptides	1.1±0.8 min	32±9 min
Low MW antibody-fragments	9.0±8.5 min	212±77 min
High MW antibody-fragments	30±238 h	10.2±2.7 h
Antibodies	17±84 h	16.8±11.8 h

## 4 Discussion

The goal of this work was to develop and disseminate a mathematical framework that can be used broadly to optimize FGS clinical protocols for individual patients. As a preliminary step, simulated PA and SA dynamics and tumor discrimination metrics (AUROC and CVR) were compared directly with human tumor xenograft mouse experiments for three imaging agent “classes:” peptide-based, affibody-based, antibody-based. The correlations between simulation and experimental parameters were all statistically significant [Fig. 6], highlighting the potential of the proposed mathematical framework to elucidate questions of, for instance (1) how much dose to give, (2) when to image, (3) how to image, and (4) how reliable are the images, without requiring costly and time-consuming trial-and-error experimentation. Moreover, the model can be tuned to the specific physiology of an individual patient, such that an FGS protocol (dose to give, timing of injection prior to surgery, etc.) could be tuned on a patient-by-patient basis. It should be noted that the experimental antibody data was only collected for 10 h; yet imaging time is typically much more delayed clinically. In these experiments, animal protocol limitation held the measurements to 10 h; however, this is still valuable for validating the mathematical framework since much of the temporal dynamics occur early after imaging agent administration. After 10 h, temporal dynamics are relatively stable. Future clinical studies with longer data collection will allow the model to be fully adapted to human conditions. Another potential limitation in this work is the absence of cellular internalization in the model. All three classes of imaging agents evaluated here are known to exhibit some level of internalization;^37^ however, it should be noted that under first-order rate constant assumption, inclusion of intracellularization in the model is mathematically identical to adding a constant to the dissociation rate $k_{\text{off}}$ (i.e., apparent $k_{\text{off}}=k_{\text{off}}+k_{\text{internalization}}$). Since the studies from which the range of $k_{\text{off}}$ was determined did not account for internalization, it is likely that the apparent $k_{\text{off}}$ was the parameter used in simulations. Future work with this model will include more nuanced evaluations of the effects of internalization in cases where first-order kinetics cannot be assumed.

The main parameters required by the mathematical framework include $K_{1}$ (extravasation rate constant), $k_{2}$ (tissue efflux rate constant), and $B_{\text{avail}}$ (level of expression of targeted biomolecule in region-of-interest versus background). In general, each of these parameters can be clinically measurable. $K_{1}$ and $k_{2}$ can be estimated from CT or MR perfusion scans,^13^^,^^38^ where these parameters are sometimes directly measured or often reported in terms of blood flow (F) and vascular permeability (permeability-surface area product, PS), where $K_{1}=F({1-e}^{-PS/F})$, and $k_{2}=\lambda PS$, where λ represents the tissue-blood partition coefficient (also measurable^36^). The targeted biomolecule concentration, $B_{\text{avail}}$, can be difficult to estimate on a patient-by-patient basis, but could be roughly determined by tissue biopsy (our group has demonstrated a correlation between $B_{\text{avail}}$ and immunofluorescence^30^). Alternatively, an average $B_{\text{avail}}$ from a tumor type could be assumed. It should be noted that determination of optimal timing is not affected by $B_{\text{avail}}$; this parameter only affects the magnitude of contrast, which could be more important for dosing concerns, rather than timing. With respect to the plasma kinetic parameter, in most cases, it is likely possible to assume a population average; however, it is quite feasible to directly measure the blood concentration of a fluorophore as a function of time on a patient-by-patient basis, noninvasively, through pulse dye densitometry^39^^,^^40^ for instance to estimate β. This could be particularly relevant for patients with diseases or less effective organs of filtration (e.g., liver or kidneys depending on the route of plasma elimination).

Note that while the magnitude of CVR is highly dependent on imaging agent dose, brightness, and sensitivity of the imaging system being used—factors not included in the analytical model of time of maximum CVR—all of these factors only affect the scale but not the shape of the CVR time curves. Therefore, they will not affect the time at which the maximum CVR is achieved, only what the maximum CVR is. While the present clinical model could be adapted to include such scaling factors, it is valuable for investigators to estimate time of maximum CVR, such that they can scale their specific “system” (imaging agent selection, dose, imaging system, etc) accordingly to achieve the CVR required for the application. Dose will affect temporal dynamics of CVR under receptor saturating conditions;^26^ however, our simulations estimate that even at 100% saturation, the error in estimated time to maximum CVR was not larger than the range of CVR about 98% of the maximum CVR (results not shown).

Another key finding in this study was the demonstration that for all tested applications (experimental and simulation), PA FGS methods provided improvements in tumor discrimination. The PA parameter of ratiometric binding potential ($BP_{\text{Ratio}}$) is roughly proportional to the number of the cancer cell surface receptors expressed, allowing for variability in tumor and background physiology (leading to nonspecific retention or variable delivery of imaging agents) to be accounted for. This helps reduce the image variability and improve CVR, which is directly linked to the discrimination statistic of the AUROC curve,^31^ a parameter recognized as directly related to how accurately a metric can be used for discrimination (at least in terms of an “ideal observer”).^41^ Moreover, the PA CVR was at least about twice as stable as the SA CVR (i.e., remained at >98% of the maximum CVR for at least twice as long as the SA CVR). This extended range of high CVR coincided with earlier achievement of high CVR for the PA compared to the SA methods, which may add further benefit by reducing the amount of time needed between imaging agent injection and surgery. PA methods have not been tested clinically to date, generally owing to the added complexity of approving multiple imaging agent administration to humans; however, there are clear advantages, which may allow higher tumor discrimination accuracy at overall lower imaging agent doses, and at earlier time points post-agent injection (same day injection and imaging). It is also noted that a first PA clinical trial is currently being planned at Dartmouth College with the ABY-029 (eIND 122681), an anti-EGFR affibody molecule that is similar to the one described here, developed in connection with LI-COR Biosciences, Inc. and affibody AB.

## 5 Conclusions

This work presents and validates mathematical frameworks to predict tumor contrast following imaging agent injection in FGS. Moreover, from these mathematical frameworks, simplistic analytical expressions estimating the optimal times for carrying out FGS after fluorescent agent administration were derived and validated for both SA and PA imaging. These results were particularly valuable considering the variance in the error of the mathematical estimate was only ∼10%, whereas patient population variations are expected to be >100% (Table 2); therefore, these estimates of optimal surgery time that can be informed by preoperative imaging could yield patient-specific improvement in tumor contrast over population-optimized surgical timing carried out in current clinical protocols.
