# Supplementary material for: Prediction of optimal contrast times post-imaging agent administration to inform personalized fluorescence-guided surgery
Source: J Biomed Opt. 2020 Nov 16;25(11):116005. doi: 10.1117/1.JBO.25.11.116005 (PMC7667427; doi:10.1117/1.JBO.25.11.116005)
Supplement: Supplementary file 1 [file JBO_025_116005_SD001.pdf]

Supplemental

Approximation of time to maximum contrast-to-variance ratio (CVR) in single-agent imaging

For simplicity, the plasma clearance is assumed to follow a mono-exponential decay such that the concentration of a cell-surface receptor targeted imaging agent in a tissue containing the specific receptor (e.g., a tumor) can be represented as a function of time, *t*, by:

$$C_T(t) \cong K_1 e^{-\beta t} \otimes e^{-k_{2a}t}; k_{2a} = \frac{k_2}{1+BP}, \tag{S1}$$

where C_T is the concentration of the imaging agent in "tumor" tissue, K_1 represents the rate constant governing extravasation of the imaging agent from the blood plasma to the tumor tissue, β is the exponential "elimination" decay constant of the imaging agent in the blood, k_2 represents the rate constant governing the efflux the imaging agent from the tumor tissue to the blood plasma, *BP* is the binding potential, and the mathematical operator represents convolution.

The concentration of the same imaging agent in a tissue devoid of targeted receptor ($BP = 0$) can then be represented as:

$$C_N(t) \cong K_1^{'} e^{-\beta t} \otimes e^{-k_2^{'}t}, \tag{S2}$$

where C_N in this "normal" tissue, and $K_1^{'}$ and $k_2^{'}$ represent the extravasation and efflux rate constants of the imaging agent in the normal tissue.

It is then possible to find an analytical solution to the convolution terms in Eqs. (S1) and (S2) using the following identity:

$$e^{at} \otimes e^{bt} = \frac{e^{at} - e^{bt}}{a-b}. \tag{S3}$$

If signal is shot noise limited (common in fluoresence), noise can be approximated as the square root of the signal intensity. As a corollary, maximum CVR and maximum signal difference

between tumor and normal tissue will be equivalent. Representing this contrast in terms of Eqs. (S1)-(S3) yields:

$$C_T - C_N = \left(\frac{K_1}{k_{2a} - \beta} - \frac{K_1'}{k_2' - \beta}\right)e^{-\beta t} - \frac{K_1}{k_{2a} - \beta}e^{-k_{2a}t} + \frac{K_1'}{k_2' - \beta}e^{-k_2' t}. \quad \text{(S4)}$$

To find the time at which Eq. (S4) reaches a maximum, t is solved for at the point where the derivative of Eq. (S4) equals zero:

$$\frac{d(C_T - C_N)}{dt} = 0. \quad \text{(S5)}$$

Rearranging the derivative of Eq. (S4) yields:

$$\beta\left(\frac{K_1'}{k_2' - \beta} - \frac{K_1}{k_{2a} - \beta}\right)e^{-\beta t} = \frac{k_{2a}K_1}{k_{2a} - \beta}e^{-k_{2a}t} - \frac{k_2' K_1'}{k_2' - \beta}e^{-k_2' t}. \quad \text{(S6)}$$

Dividing both sides by $e^{-\beta t}$ yields:

$$\beta\left(\frac{K_1'}{k_2' - \beta} - \frac{K_1}{k_{2a} - \beta}\right) = \frac{k_{2a}K_1}{k_{2a} - \beta}e^{(-k_{2a}+\beta)t} - \frac{k_2' K_1'}{k_2' - \beta}e^{(-k_2'+\beta)t}. \quad \text{(S7)}$$

Assuming that $\beta << k_{2a}, k_2'$ (i.e., imaging agent distribution to tissue rates are much faster than agent elimination from the blood), Eq. (S7) simplifies to:

$$e^{-k2at} = \frac{K_1'}{K_1}e^{-k_2' t}, \quad \text{(S8)}$$

which further simplifies to:

$$-k_{2a}t = -k_2' t + \ln\left(\frac{K_1'}{K_1}\right), \quad \text{(S9)}$$

and the time to maximum CVR can be represented as:

$$T_{\max} = \frac{\ln(\frac{K_1'}{K_1})}{k_2' - k_{2a}}. \tag{S10}$$

Approximation of time to maximum CVR in paired-agent imaging

To get an approximation of time to the maximum CVR for paired-agent imaging, the noise component in the denominator of Eq. (8) in the main manuscript must be treated differently from that in the single-agent imaging equations (S1)-(S10). To simplify the CVR equation for paired-agent imaging, it can be assumed that the noise for a BP estimate can be represented by $BP\cdot(1/C_U+1/C_T)^{1/2}$, where C_U represents the untargeted (control) imaging agent concentration in the "tumor" tissue. Then the CVR for *BP* can be represented with the additional equivalency noted:

$$CVR_{BP} \cong \frac{C_T - C_U}{\sqrt{C_U}}; \sqrt{C_T} \cong \sqrt{C_U}, \tag{S11}$$

This comes with the assumption that *BP* in the normal tissue is equal to zero. To find the time at which CVRBP is maximum, a similar process is followed as in the previous section. Assuming that the targeted and untargeted (control) imaging agents exhibit similar K_1 values and plasma clearance, the derivative of Eq. (S11) can be simplified to:

$$\frac{2}{k_2 k_{2a}} e^{-k_{2a}t} - \frac{2}{k_2^2} e^{-k_{2a}t} + \frac{1}{k_2^2} e^{-k_2 t} - \frac{1}{k_{2a}^2} e^{-k_2 t} = 0. \tag{S12}$$

Combining like exponential terms yields:

$$(\frac{2}{k_2^2} - \frac{2}{k_2 k_{2a}}) e^{-k_{2a}t} = (\frac{1}{k_2^2} - \frac{1}{k_{2a}^2}) e^{-k_2 t}. \tag{S13}$$

And taking the natural log of both sides of Eq. (S13) yields:

$$(k_2 - k_{2a})t = [\ln(\frac{1}{k_2^2} - \frac{1}{k_{2a}^2}) - \ln(\frac{2}{k_2^2} - \frac{2}{k_2 k_{2a}})], \tag{S14}$$

which finally simplifies to:

$$T_{\max} \cong \frac{1}{k_2 - k_{2a}}[\ln(\frac{1}{k_2^2} - \frac{1}{k_{2a}^2}) - \ln(\frac{2}{k_2^2} - \frac{2}{k_2 k_{2a}})]. \quad \text{(S15)}$$

Equation (S15) is an analytical approximation of the time of maximum CVR for paired-agent imaging.

Kinetic data to test the correlation between true values and analytical approximation of time to maximum CVR and finding the CVR T_{max} maps

In the last part of the paper, analytical estimations of CVR T_{max} for single- and paired-agent imaging techniques were evaluated against simulated "truth." The input parameters for generating Fig. 7 and Table 3 in the main manuscript were taken from Schmidt and Wittrup.[35] Not all the values were explicitly measured in their study but based on the molecular weight of the imaging agents and their radii, approximated ranges for each class of imaging agent were extracted from their paper. Table S1 explicitly displays the ranges of parametric values used.

Table S1 Time of maximum tumor contrast data. The values show the minimum and maximum values of each variable for each group of imaging agent.

Parameter	MW (kDa)	Radius (nm)	Permeability (10^{-7} cm/s)	Void fraction	K_D (10^{-9} M)
Peptides	0.5 – 2	0.73 – 1	100 – 500	0.45	1 – 3
Low MW antibody-fragments	3.5 – 7	1.2 - 1.74	10 – 50	0.35	3.5 – 4
High MW antibody-fragments	15.6 – 130	2.28 – 4.37	1 – 5	0.25	10 – 200
Antibodies	150 – 300	4.62 – 6.11	0.155 – 2.82	0.075	300 – 600

MW = molecular weight.

To convert these values to the input parameters of our analytical approximations for CVR T_{max}, data from other available resources were incorporated: K_1 was estimated from blood flow (F) and vascular permeability (permeability-surface area product, PS), where $K_1 = F(1\text{-}e^{-PS/F})$ and k_2 is the product of K_1 and tissue-blood partition coefficient ($k_2 = K_1.\lambda$). Tumor blood flow and normal

tissue blood flow were approximated as 1 and 0.5 $ml.g^{-1}.min^{-1}$, respectively.[36] Surface area was approximated as 1.7 $cm^2.g^{-1}$, with the partition coefficient assumed to be 1.64.[37]
